# Structural Characterization and Antioxidant Activity of Polysaccharides from *Athyrium multidentatum* (Doll.) Ching in d-Galactose-Induced Aging Mice via PI3K/AKT Pathway

**DOI:** 10.3390/molecules24183364

**Published:** 2019-09-16

**Authors:** Liang Jing, Jing-Ru Jiang, Dong-Mei Liu, Ji-Wen Sheng, Wei-Fen Zhang, Zhi-Jian Li, Liu-Ya Wei

**Affiliations:** Department of Pharmacy, Weifang Medical University, Weifang 261053, China; 18369607956@163.com (L.J.); jjr17865639968@163.com (J.-R.J.); zwf2024@126.com (W.-F.Z.); xiaoyawfmc@163.com (L.-Y.W.)

**Keywords:** aging, *Athyrium multidentatum* (Doll.) Ching, d-galactose, polysaccharides, *PI3K/Akt* pathway

## Abstract

The purpose of this study was to characterize the polysaccharides from *Athyrium multidentatum* (Doll.) Ching (AMC) rhizome and explore the protective mechanism against d-galactose-induced oxidative stress in aging mice. Methods: A series of experiments, including molecular weight, monosaccharide composition, Fourier transform infrared (FT-IR) spectroscopy, and ^1^H nuclear magnetic resonance (^1^H NMR) spectroscopy were carried out to characterize AMC polysaccharides. The mechanism was investigated exploring d-galactose-induced aging mouse model. Quantitative real-time reverse transcription polymerase chain reaction (RT-qPCR) and western blotting assays were performed to assess the gene and protein expression in liver. Key findings: Our results showed that AMC polysaccharides were mainly composed of mannose (Man), rhamnose (Rha), glucuronic acid (Glc A), glucose (Glc), galactose (Gal), arabinose (Ara), and fucose (Fuc) in a molar ratio of 0.077:0.088:0.09:1:0.375:0.354:0.04 with a molecular weight of 33203 Da (Mw). AMC polysaccharides strikingly reversed d-galactose-induced changes in mice, including upregulated *phosphatidylinositol 3-kinase* (*PI3K*), *Akt*, *nuclear factor-erythroid 2-related factor 2* (*Nrf2*), *forkhead box O3a* (*FOXO3a*), and *hemeoxygenase-1* (*HO-1*) mRNA expression, raised *Bcl-2*/*Bax* ratio, downregulated *caspase-3* mRNA expression, enhanced Akt, phosphorylation of Akt (p-Akt), Nrf2 and HO-1 protein expression, decreased caspase-3, and Bax protein expression. Conclusion: AMC polysaccharides attenuated d-galactose-induced oxidative stress and cell apoptosis by activating the *PI3K/AKT* pathway, which might in part contributed to their anti-aging activity.

## 1. Introduction

Polysaccharides are polymers of sugars and play important roles in energy storage, signal transduction and as structural components in all living organisms. In recent years, polysaccharides have drawn much attention due to their beneficial properties of anti-aging, antioxidant, anticancer, anti-inflammatory, and immunomodulatory activities [1,2,3]. Evidence shows that the physicochemical properties of the polysaccharides, such as molecular weight, monosaccharide composition, and glycosidic linkage patterns, are closely related to their biological activities [4,5]. Therefore, studies on the physicochemical properties are critical to understand their biological activities.

*Athyrium multidentatum* (Doll.) Ching (AMC), an edible medicinal fern, belongs to the family Athyriaceae and widely distributes in Changbai Mountain area of China. It has attracted considerable attention due to its therapeutic potential on high blood pressure, parasites, and rheumatism, etc. Previous investigations have revealed that polysaccharides contributed to the antioxidant and anti-aging capacities of AMC rhizome, including the lowered free radical level *in vitro*, the ameliorated pathological changes in hippocampus, the adjusted organ indices, the increased interleukin-2 (IL-2) content, the decreased tumor necrosis factor-α (TNF-α) and malondialdehyde (MDA) levels, and the augmented antioxidants in d-galactose-induced aging mice, and showed good prospects for the commercial development of functional food and medicine [6,7]. However, there is a lack of systematic investigation on the protective mechanism of AMC polysaccharides against d-galactose-induced oxidative stress.

Aging is a process of gradual functional decline at the cellular or organismal levels and considered as a chief risk factor for the progress of age-related diseases, including atherosclerosis, osteoporosis, renal failure, Alzheimer’s and Parkinson’s diseases [8,9]. It can be aggravated by various cellular stressors such as telomere erosion, DNA damage, oxidative stress or oncogenic activation. Scientists have been trying to interpret the underlying mechanisms to prolong human lifespan. However, it is still not fully understood in that a variety of signalling pathways and cellular senescence markers are involved in the process. It is generally recognized that excessive d-galactose may induce the formation of reactive oxygen species (ROS) via oxidative metabolism and advanced glycation end products and accelerate the aging process [10]. Chronic administration of d-galactose for a period of 6–10 weeks can cause deterioration of cognitive and motor function in mice, therefore, is accepted as a model of accelerated aging [11].

In this study, the structure of AMC polysaccharides was characterized by molecular weight and monosaccharide composition analyses, FT-IR and ^1^H NMR spectra. The anti-aging mechanism of the polysaccharides was explored using d-galactose-induced aging mouse model. Our study attempted to provide fundamental information on the structure characterization and reveal the underlying mechanism of AMC polysaccharides against d-galactose-induced oxidative stress in mice.

## 2. Results and Discussion

### 2.1. Preparation and Characterization of AMC Polysaccharides

**Figure 1:**
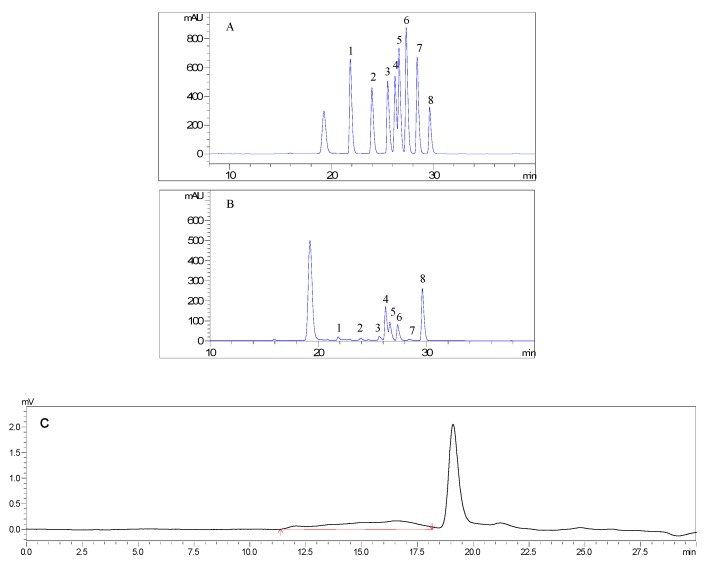
HPLC results of monosaccharide composition analysis of the standards (**A**) and the polysaccharides from *Athyrium multidentatum* (Doll.) Ching (**B**), and result of molecular weight analysis (**C**). 1, Man; 2, Rha; 3, Glc A; 4, Glc; 5, Gal; 6, Ara; 7, Fuc; 8, Rib.

AMC polysaccharides were obtained at a yield of 2.84% (calculated by weight of AMC polysaccharides/weight of AMC rhizome). The calibrated curve for molecular weight of AMC polysaccharides was given as follow logMw = −2.3696x + 19.2206. The peak retention time was from 11.367 to 18.158 min. As described in Table 1, AMC polysaccharides were heteropolysaccharides and mainly composed of Man, Rha, Glc A, Glc, Gal, Ara, and Fuc in a molar ratio of 0.077:0.088:0.09:1:0.375:0.354:0.04 with the estimated weight average molecular weight (Mw) of 33203 Da (Figure 1A–C). Polysaccharides with different monosaccharide compositions differ in their biological activities and therapeutic capacities. High level of uronic acid was reported to display significant antioxidant activity due to the presence of carboxyl groups, which acted as efficient electron or hydrogen donor in the antioxidant activity [12]. Man, Ara and Gal were strongly associated with macrophage stimulatory activities. Ara, Fuc, and Rha played important roles in radical-scavenging abilities [13]. In contrast, Glc A, Gal, Ara, Rha, and Fuc might contribute to the antioxidant activity of AMC polysaccharides, and Glc as the major component might form the backbone [7,14]. 

**Figure 2:**
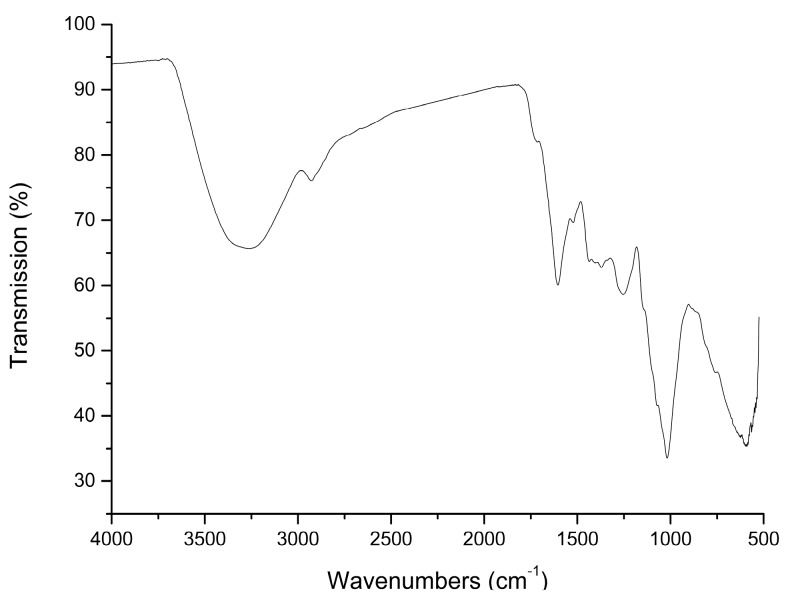
Spectrum of the polysaccharide from *Athyrium multidentatum* (Doll.) Ching.

The characteristic absorption peaks in IR spectra are very useful in identifying sugar types, sugar ring configurations and anomeric carbon atoms [15]. The IR spectrum of AMC polysaccharides was presented in Figure 2. A broad band centered at 3306 cm^−1^ was assigned to hydroxyl group and a weak band at 2925 cm^−1^ showed the C–H stretching vibration. The absorbance at 1715 cm^−1^ indicated the presence of uronic acids. The broad band at 1604 cm^−1^ was caused by -OH flexural vibrations. The bands at 1018, 1071 and 1143 cm^−1^ corresponded with C-O (C–O–C and C–O–H) stretching vibrations. The groups of bands extended from 1160 cm^−1^ to 950 cm^−1^ were assigned to O–H flexural vibrations in the pyran ring. The weak band at 888 cm^−1^ was due to the β-type glycosidic bond [16]. The weak bands at 870 and 810 cm^−1^ were induced by the characteristic peaks of mannose. 

**Figure 3:**
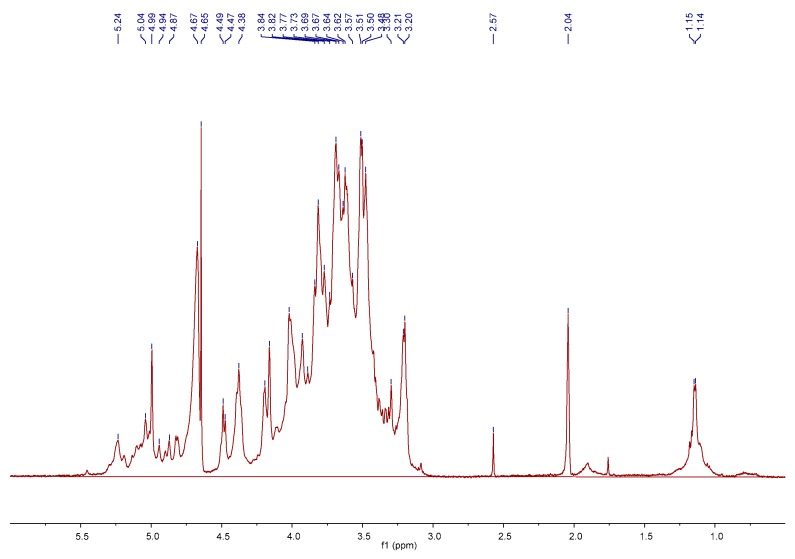
NMR spectrum of the polysaccharides from *Athyrium multidentatum* (Doll.) Ching.

^1^H NMR spectrum was employed to further confirm the obtained structure data and provide more detailed polysaccharide structural information. As shown in Figure 3, the ^1^H NMR spectrum displayed typical polysaccharide signals in the fields of 3.1–5.5 ppm and all of the relevant signals occurred in four regions. The anomeric regions *δ* 5.0–5.5 were designed for *α*-anomers and *δ* 4.30–4.99 were characteristic of β-anomers, which meant the linkages among the sugar residues belonged to the α- and β-type of glucosidic bonds. Ten obvious chemical shifts of anomeric protons were found at 5.24, 5.04, 4.99, 4.94, 4.87, 4.67, 4.65, 4.49, 4.47 and 4.38 ppm in the ^1^H NMR spectrum. These signals testified that AMC polysaccharides were equipped with at least ten types of units. However, only seven monosaccharides were found to be present in the sample by PMP-HPLC analysis. The result suggested that the units might be divided into different units with different linkages. The ring proton regions *δ* 3.1–4.2 showed overlapping peaks and were attributed to the C–2 to C–6 positions of all sugar residues. The high field regions *δ* 1.0–1.2 were contributed to the methyl groups of the glycosyl linkages of rhamnose residues [17]. 

Based on these data, AMC polysaccharides are proposed to be acidic heteropolysaccharides consisting of seven monosaccharides with ten types of units and α-/β-type of glucosidic bonds. The types of side chains and the backbone units play important roles in the biological activities of polysaccharides. β(1→3) and β(1→6) linkages in the repeating units were reported to be crucial for the anticancer activity [18]. Polysaccharides with a branched (1→3)-β-d-glucan moiety from Ganoderma lucidum showed anti-apoptotic effect on neutrophils by activating Akt-regulated signalling pathways [19]. Therefore, the monomer compositions of AMC polysaccharides would be further recognized according to the analysis of partial acid hydrolysis, periodate oxidation, Smith degradation, ^13^C NMR, HSQC, and COSY spectra.

### 2.2. RT-qPCR and Western Blotting Analyses 

**Figure 4:**
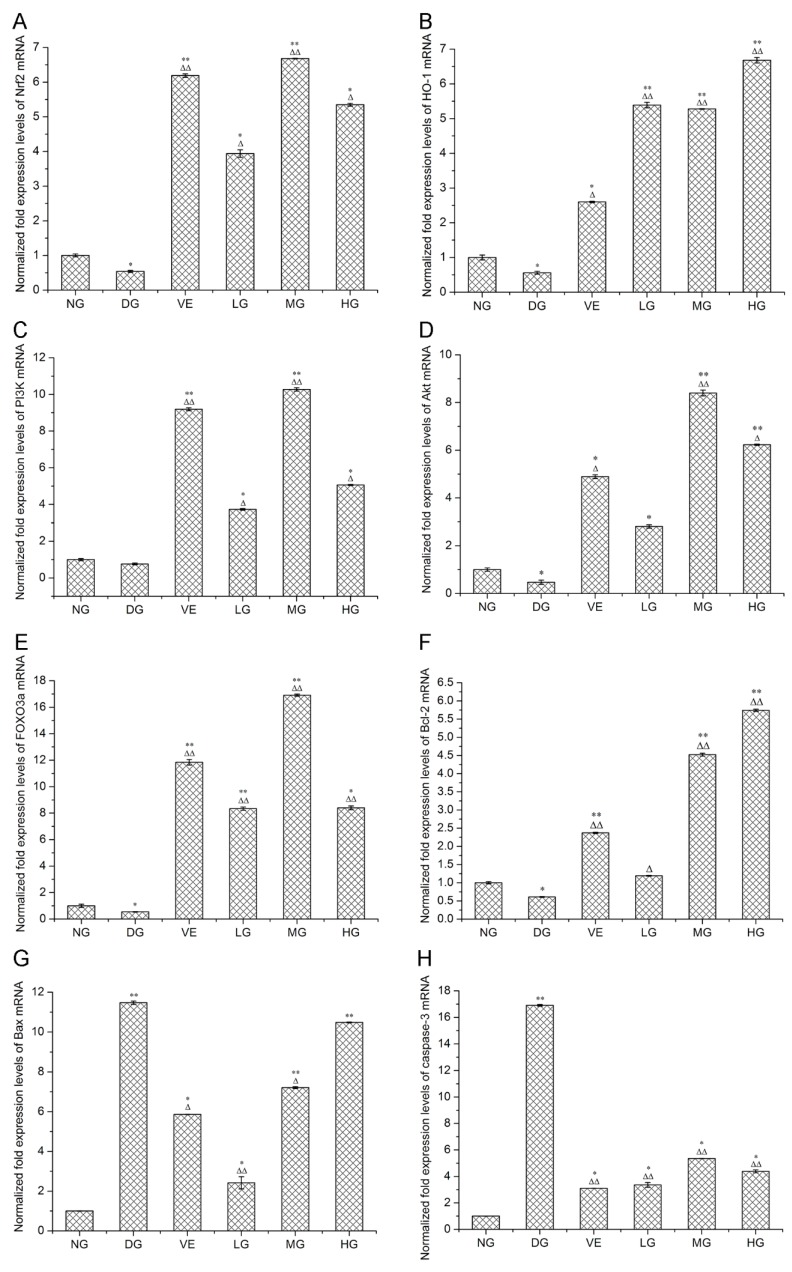
Effect of *Athyrium multidentatum* (Doll.) Ching (AMC) polysaccharides on (**A**) *Nrf2*, (**B**) *HO-1*, (**C**) *PI3K*, (**D**) *Akt*, (**E**) *FOXO3a*, (**F**) *Bcl-2*, (**G**) *Bax*, (**H**) *caspase-3* gene expression in mouse liver (n = 6). * *p* < 0.05, ** *p* < 0.01, compared with the normal group (NG); ^∆^ *p* < 0.05, ^∆∆^ *p* < 0.01, compared with the model group (DG).

**Figure 5:**
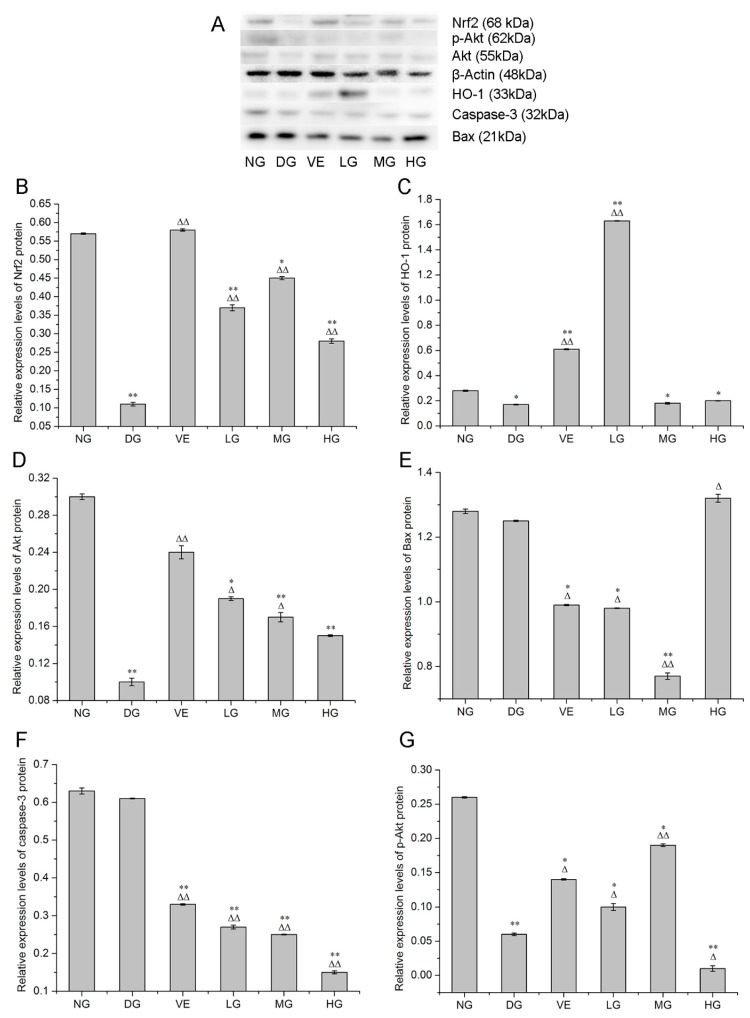
Blotting detection (**A**). Effect of AMC polysaccharides on (**B**) Nrf2, (**C**) HO-1, (**D**) Akt, (**E**) Bax, (**F**) caspase-3, (**G**) p-Akt protein expression in mouse liver (n = 6). * *p* < 0.05, ** *p* < 0.01, compared with the normal group (NG); ^∆^ *p* < 0.05, ^∆∆^ *p* < 0.01, compared with the model group (DG).

Accumulating evidence proposes that superfluous d-galactose administration is associated with increased oxidative stress, decreased antioxidants and raised cell apoptosis, which are closely associated with aging [20]. Our results showed that AMC polysaccharides treatment protected mouse liver against d-galactose-induced oxidative stress through regulating multiple intracellular redox-sensitive signaling pathways. HO-1, one of the crucial antioxidants, plays an essential role in defense against oxidative stress. Nrf2 is a major stress-response transcription factor known for its cytoprotective function. Under oxidative stress conditions, Nrf2 dissociates from Kelch-like ECH associated protein 1 (Keap1) and translocates into the nucleus, inducing the enhancement of the phase II enzyme activities such as superoxide dismutase (SOD), catalase (CAT), glutathione peroxidase (GSH-Px), HO-1 and the anti-apoptotic protein Bcl-2 [21,22]. RT-qPCR and western blotting analyses revealed that AMC polysaccharides treatment enhanced the expression of *Nrf2* and *HO-1* in mouse liver in the dose range of 100 to 300 mg/kg/d. As shown in Figure 4A,B, the expression levels of *Nrf2* and *HO-1* mRNA were declined in the model groups in comparison with the normal groups. AMC polysaccharides treatment remarkably enhanced *Nrf2* and *HO-1* mRNA expression compared with the model groups. At the dose of 200 mg/kg/d, AMC polysaccharides exhibited a more powerful effect on *Nrf2* mRNA expression (6.68 ± 0.01, *p* < 0.01) than vitamin E (6.19 ± 0.05, *p* < 0.01). *HO-1* mRNA expression levels in the three polysaccharide groups were respectively 5.39 ± 0.08, 5.28 ± 0.007 and 6.68 ± 0.08, which were much higher than the positive control group (2.60 ± 0.02). Furthermore, western blotting analysis exhibited significant alterations of Nrf2 and HO-1 protein expression. As displayed in Figure 5B, the expression level of Nrf2 protein was decreased to 0.11 ± 0.005 (*p* < 0.01) after supplying with d-galactose for 50 days. AMC polysaccharides augmented the Nrf2 protein level to 0.45 ± 0.004 at the dose of 200 mg/kg/d, which was higher than the low- (0.37 ± 0.008) and the high- (0.28 ± 0.006) dose groups, but lower than the positive control group (0.58 ± 0.003, *p* < 0.01). Likewise, d-galactose treatment significantly decreased HO-1 protein expression but AMC polysaccharides enhanced HO-1 expression (Figure 5C). The expression levels of HO-1 protein in the three polysaccharide groups were respectively 1.63 ± 0.002, 0.18 ± 0.006 and 0.20 ± 0.002 (*p* < 0.01 or 0.05). At a dose of 100 mg/kg/d, AMC polysaccharide exhibited a stronger promotion effect on HO-1 protein than vitamin E (0.61 ± 0.004). Our previous report demonstrated that AMC polysaccharides treatment led to an increase in SOD and CAT activities [6]. These results implied that AMC polysaccharides supplementation enhanced multiple antioxidants, including HO-1, SOD, and CAT by activating Nrf2 pathway, which might partly contribute to the protective effect of the polysaccharides against d-galactose-induced oxidative stress in mice.

PI3K/Akt plays a crucial role in regulating cell survival and apoptosis, which makes it a potential target of drug therapy [23]. Activation of PI3K results in the recruitment of the downstream target Akt to the plasma membrane, where it is activated by the 3-phosphoinositide-dependent kinase PDK1. The activated Akt affects the activities or abundance of a number of transcription factors linked to cell survival and cell cycle [24]. For example, the nuclear translocation of Nrf2 directly relies on the activation of the upstream PI3K/Akt signalling pathway [21]. FOXO3a, one of the most important members associated with aging in FOXO family, is an important molecule in PI3K/Akt-mediated signal transduction for regulating oxidative stress, apoptosis, longevity and cell cycle regulation [25]. In our experiments, d-galactose treatment significantly decreased the levels of *PI3K*, *Akt*, and *FOXO3a*, on which AMC polysaccharides supplementation showed effective ameliorations. At the dose of 200 mg/kg/d, AMC polysaccharides exhibited the most powerful effects on *PI3K*, *Akt*, and *FOXO3a* mRNA expression (Figure 4C–E). The values were respectively 10.27 ± 0.09, 8.4 ± 0.12 and 16.91 ± 0.08 with significant differences (*p* < 0.01). Correspondingly, the expression levels of *PI3K*, *Akt*, and *FOXO3a* mRNA were separately 9.19 ± 0.08, 4.89 ± 0.08 and 11.84 ± 0.21 in the positive control groups, which were lower than the medium-dose polysaccharide groups. Simultaneously, the expression level of the Akt protein was attenuated to 0.10 ± 0.004 after d-galactose administration (Figure 5D). The difference was significant as compared with the normal control group (0.30 ± 0.003, *p* < 0.01). In the polysaccharide groups, Akt protein levels were increased with a descending order of 0.19 ± 0.002, 0.17 ± 0.005 and 0.15 ± 0.001 (*p* < 0.01 or 0.05), suggesting that *PI3K*, Akt, and *FOXO3a* activities was inhibited by d-galactose treatment but retrieved by AMC polysaccharides supplementation. Taken together, *PI3K*/Akt/*FOXO3a* pathway is involved in the D-galactose-induced aging process and served as another mechanism against oxidative stress and apoptosis. Therefore, AMC polysaccharides may show protective effect via activating *PI3K*/*Akt* pathway and enhancing expression of its downstream targets and/or factors such as *FOXO3a* and *Nrf2*.

It is reckoned that over-expressed Bcl-2 is resistant to cell apoptosis but Bax promotes cell death by forming a heterodimer with Bcl-2 and blocking its anti-apoptotic actions [26]. Therefore, the Bcl-2/Bax expression ratio is regarded as a key determinant of a cellular fate. Caspase-3, the downstream of the Bcl-2 protein, can cleave Bcl-2 and trigger cell apoptosis when it is activated. In our experiments, reduced *Bcl-2/Bax* ratio and increased caspase-3 activity were found in the d-galactose-induced aging mice, which meant d-galactose-induced cell apoptosis [20]. As expected, increased *Bcl-2/Bax* ratio and reduced caspase-3 activity presented after treatment with AMC polysaccharides. The *Bcl-2*, *Bax* and *caspase-3* mRNA expression levels in the medium-dose polysaccharide groups were respectively 4.52 ± 0.04, 7.21 ± 0.05 and 5.35 ± 0.01 with a statistical difference *p* < 0.05 or *p* < 0.01 in contrast with the normal or the model group (Figure 4F–H). The mRNA ratios of *Bcl-2/Bax* were separatively 49.17, 62.69 and 54.77% in the three polysaccharide groups, which were higher than the model group (5.32%) and the positive control group (40.44%). The expression levels of Bax protein in the liver tissues of the mice were displayed in Figure 5E. The protein levels in the low- and medium-dose polysaccharide groups were decreased to 0.98 ± 0.001 and 0.77 ± 0.01 with statistical differences *p* < 0.05 or *p* < 0.01 in comparison with the normal or the model group, which were lower than the positive control group (0.99 ± 0.002). However, there was no dose-effect relationship. As depicted in Figure 5F, the caspase-3 protein expression levels in the low-, medium- and high-dose polysaccharide groups were respectively 0.27 ± 0.005, 0.25 ± 0.001 and 0.15 ± 0.004 with statistically significant differences (*p* < 0.01) and there existed a dose-response manner. AMC polysaccharides exerted a stronger inhibitive effect on caspase-3 protein than vitamin E. To conclude, the heightened ratio of *Bcl-2/Bax*, increased Bax protein, and reduced activity of caspase-3 gene/protein led to the decreased apoptosis, conducing to protecting against d-galactose-induced oxidative stress and aging. As a well-known apoptosis-relevant signalling pathway, *PI3K*/*Akt* might contribute to the anti-apoptotic effect of AMC polysaccharides. This hypothesis was confirmed by the elevated *PI3K/Akt* mRNA expression mentioned above and the increased p-Akt level (Figure 5G). The expression levels of p-Akt protein in the normal and model groups were respectively 0.26 ± 0.001 and 0.06 ± 0.002. The protein level in the medium-dose polysaccharide group was increased to 0.19 ± 0.002 with statistical difference *p* < 0.05 or *p* < 0.01, which was higher than the positive control group (0.14 ± 0.001). These data supported strongly that AMC polysaccharides upregulated *Bcl-2/Bax* ratio via activating *PI3K/Akt* pathway and consequently exerted its antioxidant function in d-galactose-induced aging mice. 

The mechanisms involved in senescence usually include the p53 or p16 tumour suppressor pathways. p53 is involved in the regulation of the cell cycle, DNA repair, and apoptosis [27]. Our previous studies reported that *p53* and *p21* gene expression were up-regulated in d-galactose-treated mice. AMC polysaccharides supplement remarkably decreased *p53* and *p21* activities, which was in accordance with Wu et al. [28]. p53 is upstream of FOXO3a and can crosstalk with FOXO3a. Over-activated p53 can downregulate FOXO3a expression and accelerate DNA damage-induced cell apoptosis. In our experiment, the increased expression of *FOXO3a* by AMC polysaccharides might form an inverse feedback to inhibit *p53* activity, which contributed to cell survival and anti-aging. Current results might help to understand the different roles of *FOXO3a* and *p53* in d-galactose-induced stress responses. 

## 3. Materials and Methods

### 3.1. Materials and Reagents

The *Athyrium multidentatum* (Doll.) Ching rhizome was harvested manually from Changbai Mountain area of China in October 2016 and identified by Prof. Chongmei Xu (Weifang Medical University, Weifang, China). d-galactose and vitamin E were supplied by Sigma Chemical Co. Ltd. (St. Louis, MO, USA). HiFiScript gDNA removal cDNA synthesis kit was purchased from ComWin Biotech Co. Ltd. (Beijing, China). SYBR^®^ green realtime PCR master mix was obtained from Toyobo Co. Ltd. (Osaka, Japan). *FOXO3a*, *PI3K*, *Akt*, *caspase-3*, *Bax*, *Bcl-2*, *HO-1*, *Nrf2* and *β-actin* primers were synthesized by Ruiboxingke Biotech. Co. Ltd. (Beijing, China). BCA protein assay kit, trizol reagent, PVDF film (0.22 μM), prestained protein marker (11-180 kDa), RIPA lysis buffer and skim milk powder were purchased from SolarbioScience & Technology Co. Ltd. (Beijing, China). Akt, p-Akt, caspase-3, Bax, HO-1, Nrf2 and *β*-actin polyclonal antibody (rabbit anti-mouse), and peroxidase-conjugated affinipure goat anti-rabbit IgG (H+L) antibody were acquired from Proteintech Group (Rosemont, IL, USA). SuperSignal^®^ West Pico ECL chemiluminescent substrate was purchased from Pierce Biotechnology (Rockford, IL, USA). 

### 3.2. Preparation and Characterization of AMC Polysaccharides

Crude AMC polysaccharides were prepared by the method of hot water extraction and ethanol precipitation. Briefly, 1.2 kg powdered AMC rhizome was refluxed in distilled water for two times, each time 1.5 h. Subsequently, the whole extract was filtered and concentrated to 200 mL, then precipitated with 600 mL anhydrous ethanol. AMC polysaccharides were obtained after filtration through a Buchner funnel.

The molecular weight was determined using HPLC (LC-20A, Shimadzu Corporation, Kyoto, Japan) equipped with a gel chromatography column of TSK G3000 PWxl (300 × 7.8 mm, 5 µm, TOSOH Corporation, Yokkaichi, Japan) and a refractive index detector. The injection volume was 25 μL and the mobile phase was 0.1 mol/L Na_2_SO_4_ buffer at a flow rate of 0.5 mL/min. The related calibration curve was constructed by using a series of dextrans with known molecular weights as standards. 

The monosaccharide composition was analyzed by PMP pre-column derivation method as described previously [7]. In brief, 10 mg of polysaccharide sample was hydrolyzed in a sealed glass tube with 1 mL trifluoroacetic acid (4 mol/L) at 105 °C for 4 h. The surplus acid was completely neutralized with 2 mol/L NaOH. After being adjusted to 10 mL with distilled water, the hydrolyzed products were derivatized with 0.3 mol/L NaOH and 0.5 mol/L 1-phenyl-3-methyl-5-pyrazolone (PMP) at 70 °C for 30 min, then neutralized with 0.3 mol/L HCl. Finally, the solution was extracted three times with 0.5 mL CHCl_3_. The supernatant liquor (20 μL) was analyzed by an Agilent 1260 HPLC system equipped with a YMC-Pack ODS-AQ column (250 × 4.6 mm, 5 µm, YMC, Kyoto, Japan) and detected at 245 nm. The triethylamine ammonium acetate buffer/acetonitrile (9:1, 4:6, v/v) was used as mobile phase with a flow rate of 1.0 mL/min and the column temperature was maintained at 30 °C. Seven monosaccharides, rhamnose, fucose, arabinose, mannose, galactose, glucose, and glucuronic acid, were used as standards to quantify the monosaccharide content. Ribose (Rib) was used as the internal standard. 

The FT-IR spectrum of AMC polysaccharides was recorded with a Shimadzu FTIR-8300 spectrophotometer (Shimadzu Corporation, Kyoto, Japan) scanning between 400 and 4000 cm^−1^. The sample was analyzed by grinding a mixture of the polysaccharides with dry KBr and pressing in a mould. NMR measurements were inspected with a Bruker AV-500 spectrometer (Bruker Corporation, Karlsruhe, Germany). The sample was completely dissolved in D_2_O to obtain the ^1^H NMR spectrum. The chemical shifts were expressed in *δ* (ppm).

### 3.3. Animals and Experimental Protocol

Eighty Kunming mice, male, weighing 40 ± 2 g, were purchased from Experimental Animal Center of Shandong Province, Jinan, China (Certificate No. SCXK 20140007). The animals were housed in cages under controlled conditions of a 12:12 h light/dark cycle at 25 ± 2 °C and provided food and water *ad libitum*. After an acclimatizing period of 3 days, the mice were weighted and randomly divided into six groups including normal group (NG), positive control group (VE), model group (DG), low-dose polysaccharide group (LG), medium-dose polysaccharide group (MG) and high-dose polysaccharide group (HG). The mice were treated as follows: Positive control group, intragastric administration with vitamin E at a dosage of 200 mg/kg/d; polysaccharide groups, intragastric administration with polysaccharides at dosages of 100, 200 and 300 mg/kg/d; positive control group, model group and polysaccharide groups, intraperitoneal injection with d-galactose at 100 mg/kg/d. All drugs were administered for 50 consecutive days and adjusted according to the mouse daily weigh. All experiments were performed in accordance with the guidelines of the ethics committee of Weifang Medical University (2018024). At the end of treatment, all mice were sacrificed and the livers were kept in liquid nitrogen for RT-qPCR and western blotting assays.

### 3.4. RT-qPCR Analysis

Total RNA was extracted from the minced mouse liver tissue by Trizol method. The concentration and integrity of the RNA were measured by the absorption ratio at 260/280 using a biophotometer plus spectrophotometer (Eppendorf AG, Hamburg, Germany). Total RNA was reverse transcribed into cDNA according to the manufacturer’s instructions. The mRNA expression was determined through SYBR green-based quantitative real time RT-PCR on a Lightcycler 480 II PCR instrument (Roche Diagnostics GmbH, Mannheim, Germany). The RT-qPCR protocol consisted of the following steps: 95 °C for 30 s, 40 cycles at 95 °C for 5 s, 55 °C for 10 s and 72 °C for 15 s. Sequences of the primers used in this study were listed in Table 2. 

### 3.5. Western Blotting Analysis

All the liver samples were extracted with RIPA lysis buffer and the protein concentrations were measured by BCA protein assay kit according to the manufacturer’s protocol. The resultant protein samples were used for the western blotting analysis. In brief, 30 µg of protein sample was analyzed with SDS-PAGE electrophoresis under the conditions of 5% stacking gel, 10% separating gel, and voltage 100 V for 1 h, then electroblotted onto a PVDF membrane (0.22 µm) at 250 mA for 1 h. Afterwards, the membranes were incubated in primary antibodies, including Akt (1:3000), p-Akt (1:3000), caspase-3 (1:2000), Bax (1:3000), HO-1 (1:3000), Nrf2 (1:2000) and β-actin (1:5000), at 4 °C overnight, then incubated in HRP-conjugated secondary antibodies (1:3000) at room temperature for 1 h. Finally, the protein bands were detected using a chemiluminescent HRP substrate. The images were captured using a FluorChem Q Imaging System (ProteinSimple, Shanghai, China). All data were analyzed by the software of Photoshop CS5 and the protein values were normalized against β-actin. 

### 3.6. Statistical Analysis

The data were presented as the means ± standard deviation and analyzed by one-way ANOVA followed by Dunnett’s test using SPSS software (version 19.0; SPSS, IBM Corporation, Armonk, NY, USA). A *p* value less than 0.05 or 0.01 was considered statistically significant.

## 4. Conclusions 

To summarize, d-galactose treatment-induced obvious aging-related changes, including aggravated oxidative stress, attenuated antioxidants, and heightened cell apoptosis. AMC polysaccharides showed effective anti-aging capacity by activating *PI3K/*Akt*/Nrf2* and *FOXO3a* pathways, increasing and/or inhibiting the expression of their downstream antioxidants and factors. Current findings provided us new perspectives for the protective mechanism of AMC polysaccharides. Further investigations concerned with the relationships between the structure and the protective effect of AMC polysaccharides will proceed in the future.

## Figures and Tables

**Table 1:** Weight and chemical components of the polysaccharides from *Athyrium multidentatum* (Doll.) Ching.

Molecular Weight (Mw, Da)	Molar Ratio of Monosaccharide Composition						
	Man	Rha	Glc A	Glc	Gal	Ara	Fuc
33203	0.077	0.088	0.09	1	0.375	0.354	0.04

**Table 2:** Sequences used for RT-qPCR.

Name	Primer Sequences (Forward/Reverse Primer)	Tm (°C)	GC (%)
*FOXO3a*	GGCGGCGTGCGGTCTCCATG	65.65	75
	TGCTGGCGTTGGAATTCCTG	57.45	55
*PI3K*	TAGGAGGAGGTTGGAAGAAG	55.4	50
	CGTCAGCCACATCAAGTATT	53.35	45
*Akt*	TGGCAGGATGTGTATGAG	52.62	50
	CTGGCTGAGTAGGAGAAC	54.9	55
*Caspase-3*	AGCACTGGAATGTCATCTCG	55.4	50
	TCCTTAGAAACACTATCCAT	49.25	35
*Bax*	GCATCAGGGTTTCATCCAGG	57.45	55
	AATCATCCTCTGCAGCTCCA	55.4	50
*Bcl-2*	GGACTTGAAGTGCCATTGGT	55.4	50
	CGGTAGCGACGAGAGAAGTC	59.5	60
*HO-1*	CCCAGTCTATGCCCCACTCT	59.5	90
	AGACGCTTTACATAGTGCTG	53.35	45
*Nrf2*	CAGCATAGAGCAGGACAT	52.62	50
	GGAACAGCGGTAGTATCA	52.62	50
*β-actin*	CACCACACCTTCTACAATGAG	55.61	47.62
	TACGACCAGAGGCATACAG	55.16	52.63
